# District-Level Health Management and Health System Performance: The Ethiopia Primary Healthcare Transformation Initiative

**DOI:** 10.34172/ijhpm.2020.236

**Published:** 2020-12-08

**Authors:** Lingrui Liu, Mayur M. Desai, Netsanet Fetene, Temsgen Ayehu, Kidest Nadew, Erika Linnander

**Affiliations:** ^1^Global Health Leadership Initiative, Yale University, New Haven, CT, USA.; ^2^Department of Health Policy and Management, Yale School of Public Health, New Haven, CT, USA.; ^3^Department of Chronic Disease Epidemiology, Yale School of Public Health, New Haven, CT, USA.; ^4^Federal Ministry of Health, Government of Ethiopia, Addis Ababa, Ethiopia.

**Keywords:** Management Capacity Intervention, Performance Management, Primary Care, Longitudinal Assessment, Ethiopia, Sub-Saharan Africa

## Abstract

**Background:** Despite a wide range of interventions to improve district health management capacity in low-income settings, evidence of the impact of these investments on system-wide management capacity and primary healthcare systems performance is limited. To address this gap, we conducted a longitudinal study of the 36 rural districts (woredas), including 229 health centers, participating in the Primary Healthcare Transformation Initiative (PTI) in Ethiopia.

**Methods:** Between 2015 and 2017, we collected quantitative measures of management capacity at the district and health center levels and a primary healthcare key performance indicator (KPI) summary score based on antenatal care (ANC) coverage, contraception use, skilled birth attendance, infant immunization, and availability of essential medications. We conducted repeated measures analysis of variance (ANOVA) to assess (1) changes in management capacities at the district health office level and health center level, (2) changes in health systems performance, and (3) the differential effects of more vs less intensive intervention models.

**Results:** Adherence to management standards at both district and health center levels improved during the intervention, and the most prominent improvement was achieved during district managers’ exposure to intensive mentorship and education. We did not observe similar patterns of change in KPI summary score.

**Conclusion:** The district health office is a valuable entry point for primary healthcare reform, and district- and facility-level management capacity can be measured and improved in a relatively short period of time. A combination of intensive mentorship and structured team-based education can serve as both an accelerator for change and a mechanism to inform broader reform efforts.

## Background

**Key Messages**

** Implications for policy makers**

- Our study provided strong and direct empirical evidence from a low-income country setting that district health office is a valuable entry point for primary healthcare reform.
- District- and facility-level management capacity can be measured using standardized tools and improved in a relatively short period of time.
- A combination of intensive mentorship and structured team-based education can serve as both an accelerator for change and a mechanism to inform broader reform efforts.

** Implications for the public**

Management capacity is essential for reaching global health goals. However, empirical evidence assessing the impact of management strengthening interventions is limited. Our study assessed the impact of a two-year project to build district management capacity at national scale in Ethiopia. We show that district- and facility-level management capacity can be measured and improved in a relatively short period of time, and that a combination of intensive mentorship and structured team-based education can serve as both an accelerator for change and a mechanism to inform broader reform efforts.

Management capacity is essential for effective healthcare systems, and is particularly critical to scaling up coverage of essential health services in resource limited settings.^1-6^ Prior research evaluating the role of management in improving health system performance has primarily focused on high- or upper-middle-income country settings,^7-11^ where researchers have demonstrated that management plays an important role in performance improvement at the organizational and system levels. Upon analysis of representative African country cases,^2,12^ the World Health Organization (WHO) concluded that building managerial capacity at the district level is critical to strengthening health systems in low-income countries.^1^ District-level management practice has been shown to be associated with primary care performance in cross-sectional studies.^13,14^

 However, rigorous empirical evidence from interventions to enhance management practice in low-income countries is limited.^3^ Existing evidence is largely derived from case studies describing the influence of management development program interventions on selected healthcare delivery outcome indicators as part of targeted quality improvement efforts (eg, antenatal care [ANC] visits, skilled birth attendant deliveries, or fully-immunized children), through application of problem-solving approaches such as Diagnose-Intervene-Verify-Adjust or Tanahashi bottleneck analysis in small target geographies,^15,16^ or on individual manager competencies and behaviors.^17-23^ We are not aware of any interventional study quantifying changes in organization- or system-level changes in district-level management practice in a low-income setting. Moreover, evaluation of prior district-level management interventions did not allow for incorporating varying levels of intervention model, limiting our ability to understand whether outcomes would have been sensitive to more or less resource-intensive approaches.^17,24^ Furthermore, none of the studies take a broader systems perspective to examine changes in downstream health center-level management practice or primary healthcare system performance in conjunction with district-level management intervention.

 Accordingly, we conducted a two-year longitudinal study of changes in both district and health center management, as well as concomitant changes in health system performance, in districts participating in a multi-faceted management and leadership development intervention in four regions of Ethiopia. We evaluated impact on quantifiable measures of management and leadership capacity at the organizational level. In addition, we examined health system performance using a composite score of key performance indicators (KPIs), as compared to many quality improvement efforts which target a single KPI. Finally, we investigated the differential impact of embedding intensive management mentorship and certificate-level education versus lighter support for roll-out of management tools and systems.

###  Setting

 We conducted this study in Ethiopia, the second most populous country in Africa. As Ethiopia has made impressive gains in scaling up healthcare access, the challenge has shifted to improving the performance of its primary healthcare system.^25^ In its Health Sector Transformation Plan (HSTP) 2015-2020,^26^ the Federal Ministry of Health (FMoH) declared health system strengthening and achieving safer, more effective, more accessible, and more equitable care as a national priority. One pillar of the HSTP is to strengthen primary healthcare through woreda(district) transformation.

 Following regions and zones, woredas in Ethiopia are the third-level administrative division of the country. The woreda health office links national- and regional-level leadership, where policies are formulated, to the facility- and community-level, where services are delivered. As the most frontline primary care administrative body, woreda health offices are responsible for planning, resource allocation, execution, monitoring, and evaluating of primary healthcare services. The woreda health offices supervise and coordinate primary care services for catchment areas of approximately 200 000 population, including oversight of 4-5 health centers, 20-30 health extension workers, and, in some cases, a primary hospital.^13^

## Methods

###  Intervention

 The Primary Healthcare Transformation Initiative (PTI) in Ethiopia was launched in 2015, aligning with the government’s commitment to build a culture of performance management and accountability, preparing the woreda to lead the ambitious set of reforms laid out in the HSTP. PTI was implemented by the Yale Global Health Leadership Initiative and funded by the Bill & Melinda Gates Foundation.

 The PTI approach included 5 levers of change: (1) intensive mentorship and certificate-level education in leadership and management (the Primary healthcare Management Development Program) for members of the woreda and health center management teams in each woreda, reaching 488 managers between 2016 and 2017; (2) development, testing, and refinement of a targeted set of KPIs to measure woreda-wide performance in primary healthcare; (3) development, testing, and refinement of a set of management standards at the health center and woreda health office levels to measure and promote improvements in management capacity; (4) restructuring the governance and accountability of the woreda health office in alignment with its core managerial functions; and (5) introduction of a quarterly performance review process at the woreda level where diverse stakeholders from across each woreda came together for a structured, supportive peer-review of woreda performance based on the management standards and KPIs.

 Each year, PTI sites received either “intensive” or “light touch” intervention. Under the “intensive” intervention, a PTI mentor (called a technical advisor for management systems, or TAMS) was embedded in the woreda office full-time for 12 months to deliver certificate-level education and provide ongoing mentorship and coaching to the woreda health office team in the development and implementation of the reform levers described above. Under the “light-touch” condition, woreda management teams received exposure to the tools and processes associated with the reforms described above, but without intensive mentorship or education. These “light-touch” sites were representative of the more diffuse changes driven by regional and national adoption of the PTI tools and systems.

###  Study Design and Sample 

**Figure 1:**
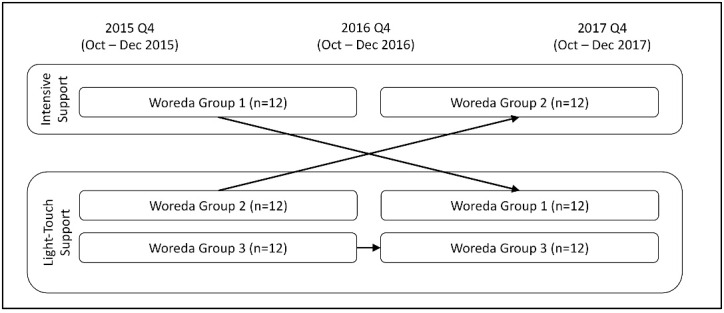
Crossover Design With a Control Group.

 As described previously,^13^ the PTI intervention focused on 36 woredas, including 229 health centers, across 4 regions of Ethiopia. Most PTI-supported woredas and their affiliated health centers were located in densely populated rural areas of the country. The woredas were divided into three groups of 12. Using a crossover design with a control group (Figure 1), the first group received intensive intervention in Year 1 (January-December 2016), followed by light-touch intervention in Year 2 (January-December 2017). The second group received light-touch intervention in Year 1, followed by intensive intervention in Year 2. The third group received only light-touch (no intensive) intervention throughout the 2-year study period. This study design provided a unique opportunity to investigate the differential impact of intensive versus light-touch intervention on management capacity and organizational performance.

###  Measures of Management Capacity 

 As reported previously,^13^ we quantified the management capacity at the woreda and health center health office levels. At the woreda level, we used the Woreda Management Standards (WMS), a regionally- and nationally-endorsed set of 26 standards in 5 domains: governance and organizational capacity, service delivery, community engagement, collaboration with other sectors, and performance management. For each woreda health office, adherence to WMS, both overall and by domain, was measured as the percentage of standards met.

 Health center management capacity was measured using the Ethiopia Health Center Reform Implementation Guidelines (EHCRIG).^13^ EHCRIG included 88 standards in 10 domains: leadership and governance, health post support, patient flow, medical records management, pharmacy services, laboratory services, infection prevention safety, medical equipment management, human resource management, and performance quality improvement. For each health center, adherence to EHCRIG was measured as the percentage of standards met.

###  Measure of Primary Healthcare Service Performance

 As reported previously,^13^ to capture overall performance of the primary care system, we generated a KPI summary score composed of 5 KPIs: (1) contraceptive acceptance rate, ie, the number of women reporting use of modern contraception divided by the estimated number of women of childbearing age who are not pregnant in the health center catchment area; (2) ANC coverage, ie, the number of women having ≥4 ANC visits divided by the number of expected births in the health center catchment area; (3) skilled birth attendance rate, ie, the number of women who give birth in a health facility divided by the expected number of births in the health center catchment area; (4) the percentage of 1-year-old children who have received all recommended immunizations in the health center catchment area; and (5) essential drug availability, ie, the average percentage of 22 essential drugs to be found in stock per month at health centers.^13^ These 5 KPIs were a subset of the 18 KPIs. As endorsed by the FMoH and Regional Health Bureaus as part of the Health Services, Development, and Planning national planning efforts, and as routinely captured in the government’s health management information system. The 5 were selected from 18 through consultation with FMOH and Regional Health Bureaus counterparts because they were most consistently reported with reliable data quality, indicated sufficient variation and room for improvement, and captured diverse aspects of system performance. For each health center, the 5 indicators, each normally distributed, were averaged to create a mean KPI summary score that could range from 0%-100%.

###  Data Collection 

 As described previously,^13^ quarterly data on adherence to the management standards and performance on the KPIs were collected from all 36 woredas (including 229 health centers) at three time points during the study period. October-December 2015 (collected in Q1 2016) represented baseline performance prior to the intervention; October-December 2016 (collected in Q1 2017) represented performance at the end of program year 1, and October-December 2017 (collected in Q1 2018) represented performance at the end of year 2. Data were collected by the 12 PTI TAMS and four PTI senior regional managers after receiving training on the data collection tool and quality control activities. Data were obtained from the woreda health office and health facility heads or their delegates using interviews with the key informants, review of relevant official documents and routine administrative data, and direct observations.

###  Statistical Analysis 

 We used standard descriptive statistics to characterize woreda and health center management capacity and performance by region and intervention group. We conducted repeated measures analysis of variance (ANOVA) to assess changes in WMS, EHCRIG, and KPI summary score over time and by intervention group. There were no missing data for WMS, and minimal missing data for EHCRIG and KPI values (<5%). Records with missing data were dropped from the longitudinal analysis of the given outcome. Analyses were performed in Stata, version 15.1, and *P*< .050 was considered statistically significant.

## Results

###  Description of Woredas and Health Centers 

 Our sample included 229 health centers in 36 woredas across four regions of Ethiopia. Regional distribution of health centers was 34% (n = 78) in Amhara; 37% (n = 85) in Oromia; 22% (n = 50) in Southern Nations, Nationalities, and People’s Region (SNNPR); and 7% (n = 16) in Tigray. Of the 36 woredas, 25% (n = 9) were in Amhara, 42% (n = 15) in Oromia, 25% (n = 9) in SNNPR, and 8% (n = 3) in Tigray.

 Table shows changes in woreda-level management capacity (WMS) and in health center-level management capacity (EHCRIG score) and performance (KPI score) both overall and by region. The average WMS score across the 36 woredas increased from 43% (standard deviation [SD] 15%) at baseline to 67% (SD 14%) at the end of the study period. The average EHCRIG score across the 229 health centers increased from 35% (SD 16%) at baseline to 59% (SD 16%) at the end of the study period. Similarly, the health center level mean KPI summary score increased from 62% (SD 22%) at baseline to 76% (SD 17%) at the end of the two-year study. Similar patterns of improvement in WMS, EHCRIG, and KPI summary scores were found in all four regions.

**Table:** Management Capacity and Performance of PTI-Supported Woredas and Health Centers in Ethiopia Over Time and by Region

**Outcome **	**Overall**			**Amhara**			**Oromia**			**SNNPR**			**Tigray**		
	**36 woreda, 229 health centers**			**9 woreda, 78 health centers**			**15 woredas, 85 health centers**			**9 woredas, 50 health centers**			**3 woredas, 16 health centers**		
	**Baseline 2015Q4**	**2016 Q4**	**2017 Q4**	**Baseline 2015Q4**	**2016 Q4**	**2017 Q4**	**Baseline 2015Q4**	**2016 Q4**	**2017 Q4**	**Baseline 2015Q4**	**2016 Q4**	**2017 Q4**	**Baseline 2015Q4**	**2016 Q4**	**2017 Q4**
Management capacity at woreda level health office: Mean (SD) WMSscore	43% (15)	58% (18)	67% (14)	42% (12)	62% (18)	67% (16)	39% (15)	53% (18)	61% (11)	47% (19)	57% (15)	73% (9)	53% (12)	71% (10)	83% (8)
Management capacity at health center: Mean (SD) EHCRIGscore	35% (16)	51% (20)	59% (16)	31% (18)	68% (13)	69% (14)	36% (13)	39% (16)	49% (13)	39% (18)	41% (16)	56% (15)	40% (12)	60% (16)	68% (10)
Health System Performance: Mean (SD) KPI summary score	62% (22)	79% (13)	76% (17)	63% (19)	65% (21)	61% (27)	44% (19)	83% (11)	77% (15)	82% (10)	70% (15)	80% (15)	68% (17)	85% (12)	76% (17)

Abbreviations: PTI, Primary Healthcare Transformation Initiative; SNNPR: Southern Nations, Nationalities, and People’s Region; SD, standard deviation; WMS, Woreda Management Standards, EHCRIG, Ethiopia Health Center Reform Implementation Guidelines; KPI, key performance indicators.

**Figure 2:**
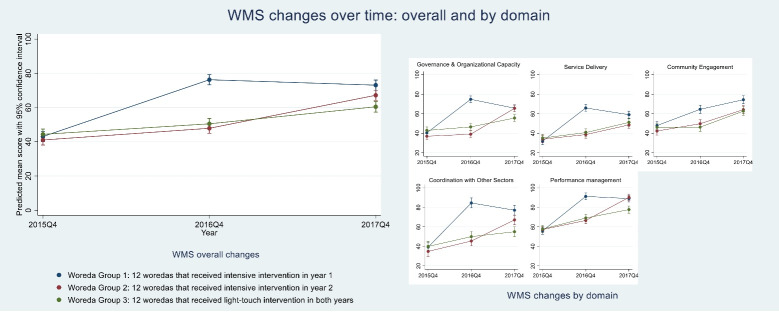
Changes in Adherence to WMS Over Time, by Woreda Intervention Group and Individual Domain. Abbreviation: WMS, Woreda Management Standards.

**Figure 3:**
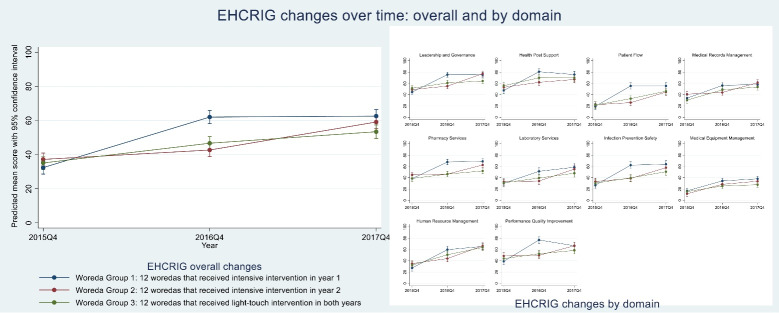
Changes in Adherence to EHCRIG Over Time, by Woreda Intervention Group and Individual Domain. Abbreviation: EHCRIG, Ethiopia Health Center Reform Implementation Guidelines.

**Figure 4:**
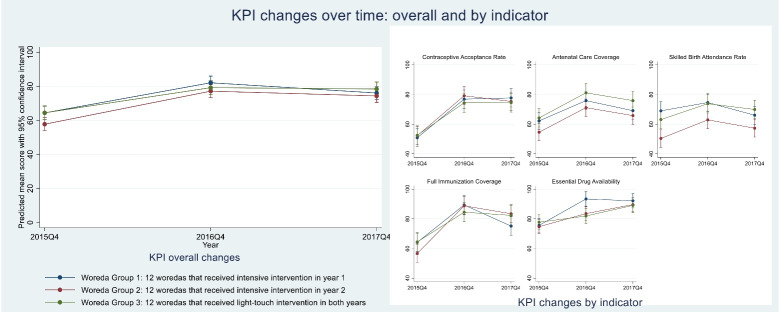
Changes in Adherence to KPIs Over Time, by Woreda Intervention Group and Individual Indicator. Abbreviation: KPI, key performance indicator.

Figures 2, 3, and 4 show results of repeated measures ANOVAs, comparing average changes in WMS, EHCRIG, and KPI summary scores, respectively, for each of the three intervention groups, thus highlighting the differential effects of intensive versus light-touch intervention. Results are also shown for the individual domains or indicators that make up the three outcome measures.

###  Change in Woreda Management Capacity

 As Figure 2 shows, all three groups had similar level of adherence to WMS at baseline. By the end of program year 1, woreda group 1, which received the intensive intervention in the first year, significantly increased its management capacity (mean WMS score increased from 43% to 76%, *P*< .001). In contrast, much more modest (not statistically significant) improvements were observed for the other two groups of woredas, which received light-touch support during the first year. During year 2, when woreda group 1 received light-touch support, its improved WMS score was sustained. However, woreda group 2, which received the intensive intervention during the second year, experienced a significant improvement (48% to 67%, *P*< .001). Woreda group 3, which received light-touch intervention throughout the two years, achieved steady improvement from baseline to Year 2 (*P*< .001), but did not reach the level of management capacity observed in the other two groups. The general patterns of improvement observed in overall adherence to WMS by woreda group were also observed for each of the five WMS domains.

###  Change in Health Center Management Capacity

 As Figure 3 presents, we observed the same general patterns of improvement in health center management capacity as those for woreda management capacity. Specifically, improvements were most pronounced during periods of intensive intervention, whereas light-touch support was associated with more modest (not statistically significant) increases or sustaining of EHCRIG scores. These patterns persisted for overall adherence to EHCRIG and for adherence within each of the 10 domains.

###  Change in KPI Performance 

 We observed a different pattern of improvement in KPI summary scores (Figure 4). Overall, the KPI summary score for each of the three woreda groups improved significantly between baseline and the end of year 1 and then plateaued between years 1 and 2. For some of the individual KPIs (ie, ANC coverage, skilled birth attendance rate, immunizations), gains achieved in year 1 seemed to decline somewhat in year 2, although not significantly so. Each individual KPI revealed different patterns of performance. With the exception of essential drug availability, the magnitude and timing of change for each of the individual KPIs was not associated with the intensity of the intervention.

## Discussion

 In this study, we sought to quantify change in management capacity and primary healthcare system performance associated with the two-year, district-level PTI intervention. We found that all three groups of woredas started at similar levels of management practice, and achieved meaningful and significant improvements in management capacity at both the woreda- and health center levels. This indicates that a district-level intervention can significantly improve management capacity at both district and health center levels.

 Significantly greater improvement in management capacity, both overall and by individual domain, was observed during periods of exposure to intensive mentorship and education. Further, in those sites receiving intensive intervention in year 1, the average improvement in management practices were sustained after the departure of the mentor. This is consistent with the PTI intervention’s focus on development of management systems, team capacity and an enabling policy environment for sustained impact,^17^ as opposed to the training of individual managers.

 By the end of the two-year intervention, woredas that received the intensive support in the first or second year achieved similar levels of management capacity. Woredas that received only light-touch support were on track to catch up over time. This was expected, given that these light-touch woredas were still exposed to the system-wide reforms PTI was supporting. These findings confirm that intensive mentorship support can be both an accelerator of early change in management practice and an essential period of learning to inform broader reform and scale up efforts, with diminishing returns over successive waves as broader reform to build national systems for healthcare performance management take hold.^27^

 Despite prior research showing a cross-sectional association between woreda management, health center management, and the KPI summary score,^13^ we did not observe concomitant changes in performance in this longitudinal study. These findings suggest that the impact of investment in management on performance may, in the short-term, be overwhelmed by other factors in the system (for example, national financing, policy, or human resources management systems),^27^ or that gains in performance may lag behind improvements in management capacity. Notably, we found similar patterns of change in availability of essential medicines over time (Figure 4), arguably the most “management sensitive” KPI component, as in WMS and EHCRIG scores. This suggests that some KPIs may be more sensitive to changes in management practice than others in the short term. This is consistent with application of the Tanahishi model in the evaluation of health services in Kenya,^28^ Ghana,^29^ which has shown that devolution efforts and vertical investments both had positive impact on availability and accessibility of essential services, but that more comprehensive approaches were needed to drive use and quality of health services.

 Our findings have several implications for policy and future research. The Sustainable Development Goals call for strengthening healthcare systems. There is global agreement that management and leadership are lynchpins in this effort. However, limited empirical evidence from low-income settings is available to guide investment and policy decisions where resources are scarce. Our study provides strong and direct evidence from a low-income country that the district health office is a valuable entry point for primary healthcare reform, that district- and facility-level management capacity can be measured and improved in a relatively short period of time, and that a combination of intensive mentorship and structured team-based education can serve as both an accelerator for change and a mechanism to inform broader reform efforts.

 To our knowledge, this is the first longitudinal study of changes in organizational management capacity in a low-income country, expanding from previous studies on the continent which have described district-level changes in individual and team problem-solving capacity through the use of targeted quality improvement models,^15-17^ and consistent with large meta-analyses from low- and middle-income country settings showing synergies between supervision, support for group problem-solving, and training.^30^ Further, our staging of exposure to intensive vs light-touch intervention enabled the investigation of the differential influences of the more intensive components of the intervention. This design, responsive to recent recommendations to replace traditional control groups with groups that receive more simple intervention models,^30^ allowed us to demonstrate that the intensive mentorship support at the district-level can be a powerful approach for accelerating improvements in management capacity while broader reforms to build a context that supports and sustains management practice take hold.^17^

 Several limitations should be noted. First, the participating districts were not randomly selected, and we did not evaluate overlap between the PTI intervention and other development partner support in any of the targeted geographies. However, they were selected in partnership with the government to achieve diversity in geography and performance, and the three groups demonstrated similar performance at baseline. Second, our observations were limited to a two-year period, and we are unable to draw conclusions about sustained impact and potential time lag between change in management and change in performance. We were, however, able to observe that Phase I woredas sustained their management practice after the mentors departed. Third, data quality can be a concern in low-resourced settings.^31,32^ In this study, however, the intervention itself had a focus on performance management, including collection and use of data for improvement, and we used explicit protocols and provided rigorous training to staff to promote data quality. We believe that the remaining data quality issues were non-differential. There are several opportunities for knowledge generation beyond the scope of this study, but ripe for future exploration. First, bottleneck analysis could be used to strengthen our understanding of supply- and demand-side factors that mitigate the relationship between improved management capacity and improved primary care system performance.^29,33,34^ Second, although we demonstrate the differential impact of the more intensive intervention, we do not present a cost-effectiveness analysis, which could help inform future investments in leadership and management capacity at scale.^30^

## Conclusion

 Our findings are consistent with the growing body of literature that calls for investment in district health offices as a lynchpin in primary health systems strengthening. We have shown that management capacity at both district and facility levels can be systematically measured and strengthened over a relatively short period of time, with intensive mentorship and education serving as a foundation for systems strengthening at national scale.

## Acknowledgement

 The authors gratefully acknowledge the leadership from each participating woreda health office and the associated health centers for their commitment to excellence; the Regional Health Bureaus and FMoH for their vision and support; and the PTI technical advisors and senior regional managers for their service as changemakers in primary care. The authors also thank Dr. Elizabeth Bradley, Jeannie Mantopolous, and Abraham Megentta for their leadership in the design and launch of PTI, and Dr. Leslie Curry for her valuable comments on this paper. Earlier versions of this paper were presented at the Africa Health Agenda International Conference 2019, where valuable comments were received from seminar participants.

## Ethical issues

 This study was exempted from human subjects review by the Human Subjects Committee of the authors institute, as it did not include individual-level protected health information.

## Competing interests

 Authors declare that they have no competing interests.

## Authors’ contributions

 Conceptualization: LL, MMD, NF, KN, EL; Data curation: LL, KN, NF; Funding acquisition: EL; Methodology: LL, MMD, EL; Project administration: NF, TA, KN; Supervision: MMD, EL; Visualization: LL; Writing-original draft: LL; Writing-review: LL, MMD, NF, TA, KN, EL.

## References

[1] World Health Organization (WHO). Towards Better Leadership and Management in Health: Report of an International Consultation on Strengthening Leadership and Management in Low-Income Countries. Geneva: WHO; 2007.

[2] Egger D, Ollier E, Tumusiime P, Bataringaya J. Strengthening Management in Low-Income Countries: Lessons from Uganda: A Case Study on Management of Health Services Delivery. Geneva: WHO; 2007.

[3] Gilson L, Agyepong IA (2018). Strengthening health system leadership for better governance: what does it take?. Health Policy Plan.

[4] Curry L, Taylor L, Chen PG, Bradley E (2012). Experiences of leadership in health care in sub-Saharan Africa. Hum Resour Health.

[5] Bradley EH, Taylor LA, Cuellar CJ (2015). Management matters: a leverage point for health systems strengthening in global health. Int J Health Policy Manag.

[6] Doherty J, Gilson L, Shung-King M (2018). Achievements and challenges in developing health leadership in South Africa: the experience of the Oliver Tambo Fellowship Programme 2008-2014. Health Policy Plan.

[7] Tangcharoensathien V, Witthayapipopsakul W, Panichkriangkrai W, Patcharanarumol W, Mills A (2018). Health systems development in Thailand: a solid platform for successful implementation of universal health coverage. Lancet.

[8] Plsek PE, Wilson T (2001). Complexity, leadership, and management in healthcare organisations. BMJ.

[9] Curtright JW, Stolp-Smith SC, Edell ES (2000). Strategic performance management: development of a performance measurement system at the Mayo Clinic. J Healthc Manag.

[10] Jack EP, Powers TL (2009). A review and synthesis of demand management, capacity management and performance in health-care services. Int J Manag Rev.

[11] Ginter PM, Duncan WJ, Swayne LE. The Strategic Management of Health Care Organizations. John Wiley & Sons Inc; 2018.

[12] World Health Organization (WHO). Managing the Health Millennium Development Goals: The Challenge of Management Strengthening: Lessons from Three Countries. Geneva: WHO; 2007.

[13] Fetene N, Canavan ME, Megentta A (2019). District-level health management and health system performance. PLoS One.

[14] Adam T (2014). Advancing the application of systems thinking in health. Health Res Policy Syst.

[15] Odaga J, Henriksson DK, Nkolo C (2016). Empowering districts to target priorities for improving child health service in Uganda using change management and rapid assessment methods. Glob Health Action.

[16] Eboreime EA, Nxumalo N, Ramaswamy R, Ibisomi L, Ihebuzor N, Eyles J (2019). Effectiveness of the Diagnose-Intervene- Verify-Adjust (DIVA) model for integrated primary healthcare planning and performance improvement: an embedded mixed methods evaluation in Kaduna state, Nigeria. BMJ Open.

[17] Kwamie A, van Dijk H, Agyepong IA (2014). Advancing the application of systems thinking in health: realist evaluation of the Leadership Development Programme for district manager decision-making in Ghana. Health Res Policy Syst.

[18] Mansour M, Mansour JB, El Swesy AH (2010). Scaling up proven public health interventions through a locally owned and sustained leadership development programme in rural Upper Egypt. Hum Resour Health.

[19] Samuels F, Amaya AB, Balabanova D (2017). Drivers of health system strengthening: learning from implementation of maternal and child health programmes in Mozambique, Nepal and Rwanda. Health Policy Plan.

[20] Heywood P, Choi Y (2010). Health system performance at the district level in Indonesia after decentralization. BMC Int Health Hum Rights.

[21] Manafa O, McAuliffe E, Maseko F, Bowie C, MacLachlan M, Normand C (2009). Retention of health workers in Malawi: perspectives of health workers and district management. Hum Resour Health.

[22] Mutale W, Vardoy-Mutale AT, Kachemba A, Mukendi R, Clarke K, Mulenga D (2017). Leadership and management training as a catalyst to health system strengthening in low-income settings: Evidence from implementation of the Zambia Management and Leadership course for district health managers in Zambia. PLoS One.

[23] Curry LA, Byam P, Linnander E (2013). Evaluation of the Ethiopian Millennium Rural Initiative: impact on mortality and cost-effectiveness. PLoS One.

[24] Conn CP, Jenkins P, Touray SO (1996). Strengthening health management: experience of district teams in The Gambia. Health Policy Plan.

[25] Fetene N, Linnander E, Fekadu B (2016). The Ethiopian health extension program and variation in health systems performance: what matters?. PLoS One.

[26] The Federal Democratic Republic of Ethiopia Ministry of Health. Health Sector Transformation Plan (HSTP) 2015-2020. Published 2015.

[27] Cometto G, Buchan J, Dussault G (2020). Developing the health workforce for universal health coverage. Bull World Health Organ.

[28] McCollum R, Taegtmeyer M, Otiso L (2019). Healthcare equity analysis: applying the Tanahashi model of health service coverage to community health systems following devolution in Kenya. Int J Equity Health.

[29] Sheff MC, Bawah AA, Asuming PO (2020). Evaluating health service coverage in Ghana’s Volta Region using a modified Tanahashi model. Glob Health Action.

[30] Rowe AK, Rowe SY, Peters DH, Holloway KA, Chalker J, Ross-Degnan D (2018). Effectiveness of strategies to improve health-care provider practices in low-income and middle-income countries: a systematic review. Lancet Glob Health.

[31] Mphatswe W, Mate KS, Bennett B (2012). Improving public health information: a data quality intervention in KwaZulu-Natal, South Africa. Bull World Health Organ.

[32] Ndabarora E, Chipps JA, Uys L (2014). Systematic review of health data quality management and best practices at community and district levels in LMIC. Inf Dev.

[33] Rupani MP, Gaonkar NT, Bhatt GS (2016). Bottleneck analysis and strategic planning using Tanahashi model for childhood diarrhea management in Gujarat, Western India. Eval Program Plann.

[34] Kiwanuka Henriksson D, Fredriksson M, Waiswa P, Selling K, Swartling Peterson S (2017). Bottleneck analysis at district level to illustrate gaps within the district health system in Uganda. Glob Health Action.

